# Emergency Medicine Physician Assistant (EMPA) Postgraduate Training Programs: Program Characteristics and Training Curricula

**DOI:** 10.5811/westjem.2018.12.41061

**Published:** 2019-02-11

**Authors:** Fred Wu

**Affiliations:** Society of Emergency Medicine Physician Assistants (SEMPA), Irving, Texas

Dear Editor:

The Society of Emergency Medicine Physician Assistants (SEMPA) read with great interest the article titled “Emergency Medicine Physician Assistant (EMPA) Postgraduate Training Programs: Program Characteristics and Curricula” by Kraus et al.^1^ We appreciate the authors conducting research describing EMPA postgraduate training program characteristics and agree that more research is needed in this field. As the largest national organization representing EMPAs, we would like to expand on a few points regarding these programs and overall EMPA practice.

Kraus at al. write that based on their research, there is an opportunity for the development of a standardized curriculum for postgraduate training programs. SEMPA also recognized this opportunity and our Postgraduate Education Committee, which is comprised of EMPA postgraduate program directors from across the country, in 2015 developed and released EMPA postgraduate training program standards.^2^ The standards are designed to serve as a guideline for new and existing programs in an effort to standardize EMPA postgraduate education.

The authors also mention that emergency physicians (EPs) have certification and re-certification exams, lifelong learning through maintenance of certification activities and that EMPAs do not have continuing education requirements. Like EPs, EMPAs have certification and re-certification requirements. We are required to pass the Physician Assistant National Certifying Examination (PANCE) offered by the National Commission on Certification of Physician Assistants (NCCPA). To maintain certification, physician assistants (PAs) are required to earn 100 credits of continuing medical education (CME) every two years and pass a re-certification exam every 10 years.^3^ SEMPA also recommends that EMPAs complete at least 50% of their CME in emergency medicine (EM).^4^

In the paper, there is a statement about how there is the lack of a specialty-specific certifying examination. Since 2011, the NCCPA has offered specialty certification in the form of an Emergency Medicine Certificate of Added Qualifications (CAQ), which requires specific CME hours, patient care experience, procedural experience, and passing an emergency medicine specialty exam.^5^

The authors state that there is voluntary accreditation through the Accreditation Review Commission on Education for the Physician Assistant (ARC-PA). Unfortunately, there is currently no accreditation process for PA postgraduate training programs. Previously, ARC-PA did accredit postgraduate training programs. However, in 2014 ARC-PA placed the accreditation process in abeyance.^6^ There has been discussion that ARC-PA may resume postgraduate training program accreditation in 2019, though nothing has been confirmed.

During their study, Kraus et al. identified 29 EMPA postgraduate training programs. Since their data collection period from October 2016 to February 2017, more programs have started and about 40 programs are currently in existence.^7^–^9^ The growth of these programs highlights the needs of the workforce along with PAs seeking more specialized training.

Finally, SEMPA would like to recognize that completing a postgraduate training program is one but not the only pathway for PAs entering EM. While Kraus et al. write that there are no EM-specific standards or competencies for EMPAs, SEMPA has previously addressed this by recommending that PAs without EM experience seek appropriate experience and education, document their learning and procedures, consider the CAQ when eligible, obtain basic certifications (ACLS, PALS, ATLS, etc.) and participate in the specialty through membership in SEMPA.^4^

Whether EMPAs complete a postgraduate program or receive on-the-job training/experience, they are valuable members of the emergency care workforce committed to partnering with EPs.
